# Multiresistant Bacteria Isolated from Intestinal Faeces of Farm Animals in Austria

**DOI:** 10.3390/antibiotics10040466

**Published:** 2021-04-20

**Authors:** Herbert Galler, Josefa Luxner, Christian Petternel, Franz F. Reinthaler, Juliana Habib, Doris Haas, Clemens Kittinger, Peter Pless, Gebhard Feierl, Gernot Zarfel

**Affiliations:** 1 D&R Institute of Hygiene, Microbiology and Environmental Medicine, Medical University of Graz, Neue Stiftingtalstraße 6, 8010 Graz, Austria; josefa.luxner@medunigraz.at (J.L.); franz.reinthaler@medunigraz.at (F.F.R.); juliana.habib@medunigraz.at (J.H.); doris.haas@medunigraz.at (D.H.); clemens.kittinger@medunigraz.at (C.K.); gebhard.feierl@medunigraz.at (G.F.); gernot.zarfel@medunigraz.at (G.Z.); 2 Institute of Laboratory Diagnostics and Microbiology, Klinikum-Klagenfurt am Wörthersee, Feschnigstraße 11, 9020 Klagenfurt, Austria; christian.petternel@kabeg.at; 3 Animal Health Service of the Department of Veterinary Administration, Styrian Government, Friedrichgasse 9, 8010 Graz, Austria; peter.pless@stmk.gv.at

**Keywords:** broiler, swine, ESBL, VRE, CTX-M, SHV

## Abstract

In recent years, antibiotic-resistant bacteria with an impact on human health, such as extended spectrum β-lactamase (ESBL)-containing *Enterobacteriaceae*, methicillin-resistant *Staphylococcus aureus* (MRSA) and vancomycin-resistant Enterococci (VRE), have become more common in food. This is due to the use of antibiotics in animal husbandry, which leads to the promotion of antibiotic resistance and thus also makes food a source of such resistant bacteria. Most studies dealing with this issue usually focus on the animals or processed food products to examine the antibiotic resistant bacteria. This study investigated the intestine as another main habitat besides the skin for multiresistant bacteria. For this purpose, faeces samples were taken directly from the intestines of swine (*n* = 71) and broiler (*n* = 100) during the slaughter process and analysed. All samples were from animals fed in Austria and slaughtered in Austrian slaughterhouses for food production. The samples were examined for the presence of ESBL-producing Enterobacteriaceae, MRSA, MRCoNS and VRE. The resistance genes of the isolated bacteria were detected and sequenced by PCR. Phenotypic ESBL-producing *Escherichia coli* could be isolated in 10% of broiler casings (10 out of 100) and 43.6% of swine casings (31 out of 71). In line with previous studies, the results of this study showed that CTX-M-1 was the dominant ESBL produced by *E. coli* from swine (*n* = 25, 83.3%) and SHV-12 from broilers (*n* = 13, 81.3%). Overall, the frequency of positive samples with multidrug-resistant bacteria was lower than in most comparable studies focusing on meat products.

## 1. Introduction

The use of antibiotics is considered to be a major factor in the development of resistance in both agriculture and human medicine. Therefore, the spread of multidrug resistant (MDR) bacteria outside the hospital environment has become a serious problem over the last years, and livestock breeding with a rather extensive use of antibiotics has turned to be a source for multiresistant bacteria.

To prevent the increasing of antibiotic resistance, an introduction of regulations have been made in recent years to reduce the use of antibiotics in agriculture. Nevertheless, even in Europe, industrial animal husbandry is not conceivable without the massive use of antibiotics [1,2,3].

Bacteria with human-induced resistance are thus regularly found on various foods, including the most important representatives in the clinical context such as extended spectrum beta-lactamase (ESBL) harbouring *Enterobacteriaceae*, methicillin resistant *Staphylococcus aureus* (MRSA) and vancomycin resistant Enterococci (VRE) [4,5,6,7,8,9].

In the last decade, the development of *Enterobacteriaceae* of ESBL phenotype was and is the most fulminant, in the clinical setting as well as in the food and environment, such as surface waters. Similar to the spread of ESBL in the healthy human population *Escherichia coli* is the most common ESBL harbouring organism in farm animals and thus also on examined foods. Notable on this resistance mechanism is the great number of animals carrying the resistance within the animal population. Most ESBL formers are found in poultry, even less in swine and little in horses and cattle. The problem with multiresistant Gram negative bacilli, is partly seen in this context as a new zoonotic pathogen [10,11,12,13,14].

The situation is different in the case of MRSA, where swine are mainly colonised with MRSA, especially LA-MRSA. This also leads to the fact that many meat products (especially pork) are contaminated with MRSA. This livestock-associated (LA)-MRSA is a clone, different from human MRSA isolates. The strains primarily found in humans are less problematic in terms of virulence, but they are still MRSA [7,15,16,17,18].

Like MRSA, vancomycin resistant Enterococci (VRE) are endemic in hospital settings and long-term care facilities and the prevalence of human colonisation is increasing [19,20]. VRE is one of the first documented antibiotic resistant bacteria with primary origin in animal farming. The rise of VRE was caused by the use of the glycopeptide avoparcin as a growth promoter starting in 1975. As avoparcin confers cross resistance to vancomycin the (mis)use of avoparcin selected for VRE until it was totally banned in animal farming in the whole European Union in 1996. However, previous studies reported a steadily decrease of VRE, but the effect of selective pressure still seems present [3,9,21,22,23]. In addition to the analysis on the individual samples, an attempt was made to establish an allocation to different herds. *Enterobacteriaceae* and Enterococci are usually present in the intestinal but normally not on skin and meat. During the slaughter process, possible multi-resistant bacteria are released from the offal of the animals and can contaminate the skin and meat of these animals.

The aim of this study was to document the presence of ESBL harbouring *Enterobacteriaceae*, VRE and methicillin resistant *Staphylococcus aureus* (MRSA) and methicillin resistant coagulase negative *Staphylococci* (MRCoNS) in the intestine content of swine and broiler, slaughtered in Austria and determination of genetic characteristics of the isolated strains. Furthermore, the study was not only to evaluate the presence of the MDR bacteria but also to see if there are differences between the individual samples of a herd or flock. 

## 2. Results

A total of 175 intestine content samples, 75 from swine and 100 from broiler, were analysed for multidrug-resistant bacteria. Of the 75 samples from swine’s intestines, 71 were included for all further analyses, the remaining four samples were excluded based on the exclusion criteria. From these samples, 71 presumptive ESBL isolates were taken and further analysed. In addition, 35 potential VRE isolates were obtained. All 100 samples from broilers could be included in the further analyses. Here, 48 presumptive ESBL isolates were obtained, as well as 35 presumptive VRE isolates. No MRSA or MRCoNS isolates were obtained either from the swine or broiler samples.

### 2.1. Swine ESBL Enterobacteriaceae 

Of the 71 intestine content samples, 21 showed ESBL positive isolates (32.4%). With regard to the slaughtered swine from 15 different herds, ESBL-forming bacteria, exclusively *E. coli*, were isolated in seven (46.6%) of them. It shows that if a herd was positive for ESBL, did not mean that all five intestine samples of the whole herd are ESBL positive. Of the 71 potential ESBLs, 30 (42.3%) distinct ESBL isolates could be genetically and phenotypically confirmed (Table 1).

All isolates were susceptible to the tested penicillin-inhibitor combinations, carbapenems, fluoroquinolones, tigecycline and amikacin. Resistance to first, second, third and fourth generation cephalosporins was clearly characteristic among the ESBL-producing *E. coli* (Table 1). High resistance rates were recorded for tetracycline in 22 (73.3%), and trimethoprim/sulfamethoxazole in 10 (33.3%) of the isolates. Resistance to nalidixic acid was detected in eight (26.7%), chloramphenicol in three (9.9%) and gentamicin in two (6.7%) of the isolates (see Supplementary Materials Table S1).

**Figure 1:**
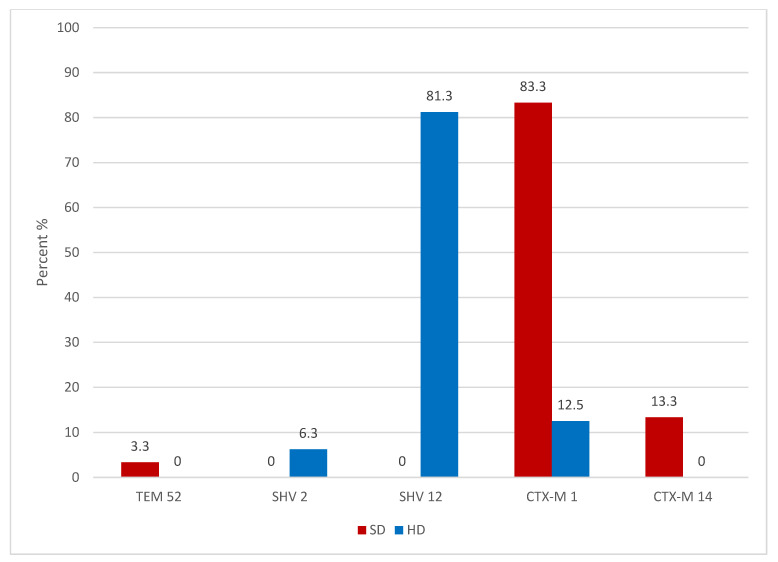
Distribution of detected ESBL members in *E. coli* isolates from swine (SD) and broiler (HD) intestine contentsamples.

CTX-M enzymes were the dominant ESBL enzyme. The most common was CTX-M-1, found in 25 (83.3%) of the *E. coli* isolates and in combination two of those harboured TEM-1 as an additional non-ESBL β-lactamase. Four (13.3%) isolates harboured CTX-M-14. From other gene families TEM-52 was found in one (3.3%) of the isolates (Table 1, Figure 1).

### 2.2. Swine VRE 

The four presumptive phenotypically VRE isolates were all found to be genotypically “false” positive, they did not show VRE resistance and no VRE genes after screening. 

### 2.3. Broiler ESBL Enterobacteriaceae

Of the 100 intestine content samples, 10 showed ESBL positive isolates (10.0%). With regard to the broiler flocks, ESBL-forming bacteria, exclusively *E. coli*, were isolated in five (50.0%) of the 10 flocks examined. Of the 35 potential ESBLs, 16 (45.7%) distinct ESBL isolates could be genetically and phenotypically confirmed. The results show that if an intestine sample from one flock was positive for ESBL, it does not mean that all intestine samples of the same flock must be ESBL positive.

All isolates were susceptible against the tested penicillin-inhibitor combinations, carbapenems, aminoglycosides and tigecycline. The isolates showed resistance to the first, second and third generation cephalosporins, in detail to cephalexin three (18.8%), cefuroxime seven (43.8%), cefotaxime 16 (100%) and ceftazidime 15 (93.8%). All isolates showed susceptibility to the cephamycin cefoxitin (Table 1). High resistance rates were recorded for tetracycline in 15 (93.8%), nalidixic acid in 14 (87.5%), chloramphenicol in 15 (93.8%) and trimethoprim/sulfamethoxazole in 10 (62.5%) isolates. From the tested fluoroquinolones low resistance was shown for moxifloxacin in three (18.8%) of the isolates whereas all isolates were sensitive for ciprofloxacin (see Supplementary Materials Table S1).

Three different ESBL genes were responsible for the ESBL resistance pattern in these 16 isolates: 13 (81.3%) isolates encoded genes for SHV-12, two (12.5%) isolates harboured genes for CTX-M-1 enzyme and one (6.3%) for SHV-2 (Figure 1). Also, the non-ESBL TEM-1 enzyme could be detected in eight (50.0%) of the isolates (seven times in combination with SHV-12 and one time with CTX-M-1) (Table 1, Figure 1). 

### 2.4. Broiler VRE 

VRE were found in two (20.0%) of the 10 broiler flocks screened. Eleven phenotypically positive isolates were detected. Each positive broiler flock had at least two intestine content samples with an average of 5.5 isolates testing positive for VRE. All isolates from each of the two flocks recovered, which were taken from the same intestine content sample showed the identical resistances pattern. They were resistant to ampicillin, vancomycin and teicoplanin (Table 1). Therefore, one *Enterococcus* isolate from each of the positive flocks were analysed in detail. Both were identified as *Enterococcus faecium* and harboured the *van*A gene. 

## 3. Discussion

Both the intensive use of antibiotics in animal husbandry and the international trade of farm animals, are important factors for the spread of livestock-associated multiresistant bacteria. Hence it is not surprising that these bacteria have also emerged in Austria, although Austria has, in contrast to other European countries, less industrialised farming and essentially smaller-sized animal farms.

The presence of *E. coli* ESBL in broiler and poultry meat products is documented by many studies from all over the world. High contamination rates (up to 93.3%) with ESBL- harbouring *Enterobacteriaceae* are reported [24,25,26].

The situation in Austria and its neighbouring countries can be assessed as less dramatic yet. A 2012 study by Springer and Bruckner, describing samples from 2009, revealed a lower ESBL frequency (35.9%) for chicken meat samples, but with a very low proportion of SHV. In contrast, recent German studies by Campos et al. and Kola et al. detected SHV-12 as the most common ESBL enzyme. Frequencies of total ESBL *E. coli* were in a similar range to the present study (45.7%), Kola et al. reported 43.9% at 2011 and Campos et al. reported 60.0% at 2012. Zelendova et al. was one of the few studies that also looked at the carcasses of pigs and found that with only 10.0% positive carcasses, the results were significantly lower than the samples from Austria [27,28,29]. 

The findings of the present study, concerning the occurrence of ESBL CTX-M-1 and SHV-12 are similar to their findings. Both genes are very common in animal farming. SHV-12 is known to be associated mainly with poultry and CTX-M-1 is widespread in mammals farm animals. The occurrence of CTX-M-15, the most common ESBL enzyme in humans, is rather rare in agriculture. Its appearance can be explained mainly by the introduction of human hosts from which the animals were colonised or the meat-products were contaminated. In contrast to other investigations, CTX-M-14 is common in farm animals and meat products and was detected with a frequency of 13.3% in swine isolates. In the present study TEM-52 was detected in swine isolates with a low frequency of 3.3% compared with other findings [25,26,28,30,31]. 

Mentioning the non ESBL co-resistance the high number of strains not susceptible to tetracycline is notable. In concordance with previous studies, this resistance is common in farm animals and meat products [32]. The rare occurrence of fluoroquinolone resistance cannot only be explained with the connection of this resistance to the appearance of CTX-M-15. Austrian reports about non-CTX-M-15 ESBL *E. coli* showed that fluoroquinolone resistance is present in human and also environmental isolates. Also, a study from Czechia revealed only 7.8% resistances to nalidixic acid of ESBL isolates from pigs [13,27,33,34]. 

Most studies focus on the investigation of meat or meat products. Colonisation with *E. coli* and other *Enterobacteriaceae* occurs primarily in the digestive tract, i.e. the meat is contaminated during the slaughter process. This can also lead to the rejection of meat from animals that do not have ESBL *E. coli* in their intestines. The data of this study also suggest that not all animals are colonised with ESBL producers and that the percentage of broiler with positive ESBL producers was lower than in meat samples from the same period and region [35]. This has clear consequences for a small-scale agriculture like Austria. Both organic and conventional animals are processed in the same slaughterhouses. The transmittance of resistant bacteria can thus easily occur among the both groups of animals. This may also explain why the difference between organic and conventional farming in Austria is not as great in terms of contamination with ESBL formers as reported from other countries with large-scale agriculture [36].

An interesting finding of this study was that even within one herd of slaughtered animals there were intestine samples containing ESBL positive isolates as well as negative isolates. Now the question arises whether there are individuals within a population contaminated with ESBL-forming germs that are less or not colonised, and whether there are individual markers that distinguish those animals from more colonised animals. In this respect, however, the conclusions of this study are also limited, since only a relatively small number of samples from a small section of the intestine were examined and the colonisation can of course vary greatly within the intestine. A follow-up study with a larger sample size and the use of modern molecular biology methods could provide further information.

MRSA and MRCoNS were not found in the intestinal samples, because obviously the skin and the nose are primary colonisation sites. In the chicken meat samples from the same period no MRSA was found and the number of meat samples from swine was also low at about 10% (including CA-MRSA) [34,35]. 

The number of VRE isolates found in this study was very low, but even after decades of the ban on avoparcin VRE are still found in animals. This is also consistent with other studies that still find VRE in animal samples, but only in very few of the samples tested. This is an example of how long the consequences of feeding (certain) antibiotics in agriculture can last. The persistence of VRE over such a long time documented in different studies all over the world, is potentially caused by co-selection with other food additives. However, it shows that a successful reset of human generated multiresistant bacteria in farming is not an easy task [9,20,22,37].

## 4. Material and Methods

### 4.1. Samples

Five intestine samples were taken from each of 15 different herds of swine (*n* = 75) and 10 intestine samples were collected from 10 different broiler flocks (*n* = 100) during the slaughtering process. The samples were collected from January to July 2012 at a slaughterhouse in the city of Graz, Austria. They were immediately transported to the laboratory under cool conditions and stored at 4 °C for processing within 24 hours.

For quality assurance only intestine content samples which showed colony forming units for coliform bacteria in the isolation and cultivation step were taken for analysis. Thus, four samples from the intestine of swine had to be excluded (*n* = 71) and all samples from the intestine of broiler (*n* = 100) were taken into account.

### 4.2. Strain Isolation and Detection

The samples from the intestine of swine were taken from the area of the midgut and the large intestine. The intestine samples were opened with a sterile scalpel and from three different segments 500 mg of digestive tract content were collected and transferred to a sterile reaction tube, 0.5 mL 0.9% NaCl solution was added and homogenised by vortexing. The preparation of the intestine samples from the broilers were first done by rinsing the intestine with one mL of 0.9% NaCl solution. The intestine was opened with a sterile scalpel and one mL of digestive tract content was transferred to a sterile reaction tube and homogenised by vortexing. Additionally, 0.1 mL from the sample solutions were used for enrichment with thioglycolate bouillon (24 h at 37 °C) for ESBL and MRSA screening. An enrichment with enterococcosel (BD Austria, Vienna, Austria) was chosen for VRE screening (24 h at 37 °C).

Afterwards decimal dilution series from the sample solutions and enterococcosel enrichment up to 10^−3^ were performed. 0.1 mL from the appropriate dilution was inoculated on chromeID^TM^ ESBL (bioMérieux, Marcy-l’Etoile, France), chromeID^TM^ VRE (bioMérieux) and OXA agar (Oxoid Ltd, Basingstoke, UK). ChromeID^TM^ ESBL agar was incubated for 24 h at 37 °C; chromeID^TM^ VRE and OXA agar for 48 h at 37 °C. Colonies were assessed as described in the manufacture´s manual, transferred to blood agar (24 h, 37 °C) and species were identified with MALDI-TOF mass spectrometry (VITEK® MS, bioMérieux). Ten isolates were taken from intestine content samples which showed colony forming units for further analysis. For enrichment sterile cotton swabs were dipped into the thioglycolate bouillon and inoculated on the selective agar plates mentioned above. Bacteria were identified as described above. 

### 4.3. Antimicrobial Susceptibility Testing

For all identified *Enterobacteriaceae*, *Staphylococcus* spp. and *Enterococcus* spp. resistance testing was performed as recommended by the European Committee on Antimicrobial Susceptibility Testing (EUCAST). The inhibition zone diameters were interpreted according to EUCAST guidelines, except tested *Enterobacteriaceae* for tetracycline, chloramphenicol and nalidixic acid, which were evaluated in conformity with CLSI guidelines (M100-S12 2011). There are no interpretation guidelines for zone diameters of these three antibiotics according to EUCAST. Specific amount of antimicrobial agents was used as follows: for *Enterobacteriaceae* ampicillin (10 µg), amoxicillin/clavulanic acid (20 µg/10 µg), piperacillin/tazobactam (100 µg/10 µg), cefalexin (30 µg), cefuroxime (30 µg), cefoxitin (30 µg), cefotaxime (5 µg), ceftazidime (10 µg), cefepime (30 µg), gentamicin (10 µg), trimethoprim/sulfamethoxazole (1.25 µg/23.75 µg), ciprofloxacin (5 µg), moxifloxacin (5 µg), imipenem (10 µg), meropenem (10 µg), tetracycline (30 µg), nalidixic acid (30 µg) and chloramphenicol (30 µg) BD BBL^TM^ Sensi-Disc^TM^ paper discs (Becton, Dickinson and Co., Sparks, MD, USA). ESBL-positive *E. coli* were confirmed by Clinical Laboratory Standards Institute (CLSI) Screening and Confirmatory Tests [38].

Antimicrobial susceptibility for *Staphylococcus* spp. was tested with penicillin (1 µg), cefoxitin (30 µg), tetracycline (30 µg), erythromycin (15 µg), clindamycin (2 µg), norfloxacin (10 µg), gentamicin (10 µg), trimethoprim/sulfamethoxazole (1.25 µg/23.75 µg), fusidic acid (10 µg), rifampicin (5 µg), linezolid (10 µg) and mupirocin (200 µg) BD BBL^TM^ Sensi-Disc^TM^ paper discs. 

Antimicrobial susceptibility for *Enterococcus* spp. was determined for ampicillin (2 µg), vancomycin (5 µg), teicoplanin (30 µg), linezolid (10 µg) and tigecycline (15 µg) by disc diffusion test, using BD BBL^TM^ Sensi-Disc^TM^ paper discs, according the EUCAST guidelines (V2.0 2012). Resistance to the glycopeptides vancomycin and teicoplanin was confirmed by ETESTt® (bioMérieux) according to the manufacturer´s instructions. *E. coli* ATCC 25922 and *Enterococcus faecalis ATCC 29212* were used as control strains in all performed tests.

### 4.4. Detection of Resistance Genes

PCR detection and gene identification were performed for three different β-lactamase gene families, *bla*_TEM_, *bla*_SHV_, *bla*_CTX-M-1group_, *bla*_CTX-M-2group_ and *bla*_CTX-M-9group_ PCR and sequencing procedures were performed as described previously by Zarfel et al. and Eckert et al. [33,39]. Taq DNA polymerase and dNTPs from QIAGEN (Hilden, Germany) were used. Sequences were compared to NCBI database. The detection of the *mec*A genes was performed as described previously by Grisold et al. [40]. The detection of the vancomycin resistance genes (*vanA/vanB)* was performed by real time PCR applying the Light cycler VRE Detection Kit (Roche, Branchburg, NJ USA) [41]. Strains with sequenced resistance genes were used as positive control for PCR, *E. coli* with *bla*_TEM-1_ and *bla*_CTXM-15_ for *bla*_TEM_, *bla*_CTX-M-1group_ PCR, *E. coli* with *bla*_SHV-12_ for *bla*_SHV_ PCR, *E. coli* with *bla*_CTX-2_ for *bla*_CTX-M-2group_ PCR and *E. coli* with *bla*_CTX-14_ for *bla*_CTX-M-9group_ PCR. 

## 5. Conclusions

The spread of multiresistant bacteria in the guts of slaughtered animals in Austria is widespread. However, this can be attributed almost exclusively to ESBL-forming *E. coli* from the examined pathogen and resistance mechanisms. In this study, intestine content of slaughtered swine and broilers tested positive for ESBL does not mean that all the samples from the same herd or flock are contaminated with multiresistant bacteria. These results showed that if herds and flocks contaminated with ESBL *E. coli*, did not mean that ESBL *E. coli* could be detected in all samples of the same herd examined. However, the detection of different ESBL genes from slaughtered animals from the same herd in different samples is probably not solely due to the sensitivity of the screening test. Individual intestinal colonisation of animals has been rather neglected in previous studies, but more data could be used to draw conclusions about which parameters lead to weaker or stronger colonisation with multidrug-resistant bacteria.

## Figures and Tables

**Table 1:** Resistance genes and antibiotic resistance profile of multiresistant bacteria isolated from the intestine content of swine and broiler.

Isolate ^a^	Species	Sample ^b^	Encoding Resistance	Resistance Pattern ^c^
SD 3/1–100a	*E. coli*	sw_01	CTX–M1	AM, CN, CXM, SXT, FEP, TET
SD 3/2–100a	*E. coli*	sw_02	CTX–M1	AM, CN, CXM, FOX, CTX, SXT, FEP, TET, NA, C
SD 3/4–100c	*E. coli*	sw_03	CTX–M1	AM, CN, CXM, CTX, CAZ, FEP
SD 3/4–100d	*E. coli*	sw_04	CTX–M1	AM, CN, CXM, CTX, SXT, CAZ, FEP, TET
SD 3/5 –100b	*E. coli*	sw_05	TEM–52	AM, CXM, CTX, SXT, CAZ, TET
SD 3/5 –100c	*E. coli*	sw_06	CTX–M1	AM, CN, CXM, CTX, FEP, TET
SD 3/5 –100e	*E. coli*	sw_07	CTX–M1	AM, CN, CXM, CTX, SXT, CAZ, FEP
SD 4/2 –100a	*E. coli*	sw_08	CTX–M1	AM, CN, CXM, CTX, GM, SXT, FEP, C
SD 4/4 –100a	*E. coli*	sw_09	CTX–M1	AM, CN, CXM, CTX, SXT, FEP, TET
SD 5/1 –100a	*E. coli*	sw_10	CTX–M14	AM, CN, CXM, CTX, TET
SD 5/1 –100b	*E. coli*	sw_11	CTX–M1	AM, CN, CXM, CTX, SXT, CAZ, FEP
SD 5/2 –100a	*E. coli*	sw_12	CTX–M1	AM, CN, CXM, CTX, CAZ, FEP
SD 5/2 –100d	*E. coli*	sw_13	CTX–M14	AM, CN, CXM, CTX, CAZ, FEP, TET
SD 5/3 –100a	*E. coli*	sw_14	CTX–M1	AM, CN, CXM, CTX, CAZ, FEP, TET
SD 5/5 –100a	*E. coli*	sw_15	CTX–M1	AM, CN, CXM, CTX, SXT, CAZ, FEP
SD 6/2 –100a	*E. coli*	sw_16	CTX–M1	AM, CN, CXM, CTX, FEP, TET, NA
SD 6/2 –100d	*E. coli*	sw_17	CTX–M1	AM, CN, CXM, CTX, CAZ, FEP, TET, NA
SD 6/4 –100a	*E. coli*	sw_18	CTX–M14	AM, CN, CXM, CTX, TET, NA
SD 6/4 –100c	*E. coli*	sw_19	CTX–M14	AM, CN, CXM, CTX, CAZ, FEP, TET, NA
SD 10/1–100b	*E. coli*	sw_20	CTX–M1	AM, CN, CXM, CTX, SXT, CAZ, FEP, TET
SD 10/4–100a	*E. coli*	sw_21	CTX–M1	AM, CN, CXM, CTX, FEP, TET
SD 10/5–100a	*E. coli*	sw_22	CTX–M1	AM, CN, CXM, CTX, GM, SXT, FEP, TET, C
SD 11/4–100a	*E. coli*	sw_23	CTX–M1	AM, CN, CXM, CTX, CAZ, FEP, TET, NA
SD 11/5–100a	*E. coli*	sw_24	CTX–M1	AM, CN, CXM, CTX, CAZ, FEP, TET, NA
SD 15/1–100b	*E. coli*	sw_25	CTX–M1	AM, CN, CXM, CTX, CAZ, FEP, TET
SD 15/2–100a	*E. coli*	sw_26	CTX–M1	AM, CN, CXM, CTX, FEP, TET
SD 15/3–100a	*E. coli*	sw_27	CTX–M1	AM, CN, CXM, CTX, FEP, TET, NA
SD 15/5–100a	*E. coli*	sw_28	CTX–M1	AM, CN, CXM, CTX, FEP, TET
SD 15/6–100a	*E. coli*	sw_29	CTX–M1	AM, CN, CXM, CTX, FEP, TET
SD 15/10–100a	*E. coli*	sw_30	CTX–M1	AM, CN, CXM, CTX, CAZ, FEP
HD 1/1 100a Th	*E. coli*	bs_31	SHV–12	AM, CXM, CTX, SXT, CAZ, TET, NA, C
HD 1/1 100b Th	*E. coli*	bs_32	SHV–12	AM, CXM, CTX, SXT, CAZ, TET, NA, C
HD 1/1 100c Th	*E. coli*	bs_33	SHV–12	AM, CXM, CTX, SXT, CAZ, TET, NA, C
HD 1/2 100a Th	*E. coli*	bs_34	CTX–M1	AM, CN, CXM, CTX, CAZ, TET, NA, C
HD 1/2 100b Th	*E. coli*	bs_35	SHV–12	AM, CN, CXM, CTX, CAZ, TET, NA, C
HD 1/2 100c Th	*E. coli*	bs_36	CTX–M1	AM, CN, CXM, CTX, SXT, MXF, CAZ, TET, NA, C
HD 1/2 100d Th	*E. coli*	bs_37	SHV–12	AM, CTX, MXF, CAZ, TET, NA, C
HD 2/9–0a	*E. coli*	bs_38	SHV–12	AM, CXM, CTX, CAZ, TET, NA, C
HD 3/2 100a	*E. coli*	bs_39	SHV–12	AM, CTX, SXT, MXF, CAZ, TET, NA, C
HD 3/3–100a	*E. coli*	bs_40	SHV–12	AM, CTX, SXT, CAZ, TET, NA, C
HD 3/4–0a	*E. coli*	bs_41	SHV–12	AM, CTX, SXT, CAZ, TET, NA, C
HD 3/5–0a	*E. coli*	bs_42	SHV–12	AM, CTX, SXT, CAZ, TET, NA, C
HD 8/2–100a	*E. coli*	bs_43	SHV–2	AM, CTX
HD 9/2–0b	*E. coli*	bs_44	SHV–12	AM, CTX, CAZ, TET, C
HD 9/2–100b	*E. coli*	bs_45	SHV–12	AM, CTX, SXT, CAZ, TET, NA, C
HD 3/10–0c	*E. coli*	bs_46	SHV–12	AM, CTX, SXT, CAZ, TET, NA, C
HD 6/1–1a	*E. faecium*	bs_47	VanA	AM, VA, TEC
HD 5/3–2a	*E. faecium*	bs_48	VanA	AM, VA, TEC

^a^ SD x/y: intestine of swine, herd number/isolate number, HD x/y: intestine of broiler, flock number/isolate number. ^b^ sw: intestine sample taken from swine, bs: intestine sample taken from broiler. ^c^ AM, ampicillin; AMC, amoxicillin/clavulanic acid; TZP, piperacillin/tazobactam; CN, cephalexin; CXM, cefuroxime; FOX, cefoxitin; CTX, cefotaxime; CAZ, ceftazidime; FEP, cefepime; CIP, ciprofloxacin; MXF, moxifloxacin; GM, gentamicin; SXT, trimethoprim/sulfamethoxazole; TE, tetracycline; NA, nalidixic acid; C, chloramphenicol; VA, vancomycin; TEC, teicoplanin.

## Supplementary Materials

The following are available online at https://www.mdpi.com/article/10.3390/antibiotics10040466/s1, Table S1: Antibiotic resistance profile and resistance genes of multiresistant bacteria isolated from the intestine of swine and broiler.
