# Supplementary material for: Multiresistant Bacteria Isolated from Intestinal Faeces of Farm Animals in Austria
Source: Antibiotics (Basel). 2021 Apr 20;10(4):466. doi: 10.3390/antibiotics10040466 (PMC8073873; doi:10.3390/antibiotics10040466)
Supplement: Supplementary file 1 [file antibiotics-10-00466-s001.zip › Table S1_30032021.pdf]

Table S1. Antibiotic resistance profile and resistance genes of multiresistant bacteria isolated from the intestine of swine and broiler.

Isolate[a]	Species	Sample[b]	Encoding resistance	Resistance pattern[c]	AM	AMC	CN	CXM	FOX	CTX	GM	SXT	CIP	MXF	TZP	CL	TGC	CAZ	FEP	IPM	AN	MEM	TE30	NA30	C30	VA	LZD	TEC
SD 3/1–100a	E. coli	sw_01	CTX–M1	am, cn, cxm, sxt, tgc, tet	R	S	R	R	S	R	S	R	S	S	S	S	S	S	R	S	S	S	R	S	S			
SD 3/2–100a	E. coli	sw_02	CTX–M1	am, cn, cxm, fox, ctx, sxt, tgc, tet, na	R	S	R	R	R	R	S	R	S	S	S	S	S	S	R	S	S	S	R	R	R			
SD 3/4–100c	E. coli	sw_03	CTX–M1	am, cn, cxm, ctx, tgc, fep	R	S	R	R	S	R	S	S	S	S	S	S	S	R	R	S	S	S	S	S	S			
SD 3/4–100d	E. coli	sw_04	CTX–M1	am, cn, cxm, ctx, sxt, tgc, fep, tet	R	S	R	R	S	R	S	R	S	S	S	S	S	R	R	S	S	S	R	S	S			
SD 3/5 –100b	E. coli	sw_05	TEM–52	am, cxm, ctx, sxt, tgc, caz, tet	R	S	S	R	S	R	S	S	S	S	S	S	S	R	S	S	S	S	R	S	S			
SD 3/5 –100c	E. coli	sw_06	CTX–M1	am, cn, cxm, ctx, tgc, fep, tet	R	S	R	R	S	R	S	S	S	S	S	S	S	S	R	S	S	S	R	S	S			
SD 3/5 –100e	E. coli	sw_07	CTX–M1	am, cn, cxm, ctx, sxt, tgc, fep	R	S	R	R	S	R	S	R	S	S	S	S	S	R	R	S	S	S	S	S	S			
SD 4/2 –100a	E. coli	sw_08	CTX–M1	am, cn, cxm, ctx, gm, sxt, tgc, fep, c	R	S	R	R	S	R	R	R	S	S	S	S	S	S	R	S	S	S	S	S	R			
SD 4/4 –100a	E. coli	sw_09	CTX–M1	am, cn, cxm, ctx, sxt, tgc, tet	R	S	R	R	S	R	S	R	S	S	S	S	S	S	R	S	S	S	R	S	S			
SD 5/1 –100a	E. coli	sw_10	CTX–M14	am, cn, cxm, ctx, tgc, tet	R	S	R	R	S	R	S	S	S	S	S	S	S	S	S	S	S	S	R	S	S			
SD 5/1 –100b	E. coli	sw_11	CTX–M1	am, cn, cxm, ctx, sxt, fep	R	S	R	R	S	R	S	R	S	S	S	S	S	R	R	S	S	S	S	S	S			
SD 5/2 –100a	E. coli	sw_12	CTX–M1	am, cn, cxm, ctx, caz, fep	R	S	R	R	S	R	S	S	S	S	S	S	S	R	R	S	S	S	S	S	S			
SD 5/2 –100d	E. coli	sw_13	CTX–M14	am, cn, cxm, ctx, fep, tet	R	S	R	R	S	R	S	S	S	S	S	S	S	R	R	S	S	S	R	S	S			
SD 5/3 –100a	E. coli	sw_14	CTX–M1	am, cn, cxm, ctx, fep, tet	R	S	R	R	S	R	S	S	S	S	S	S	S	R	R	S	S	S	S	S	S			
SD 5/5 –100a	E. coli	sw_15	CTX–M1	am, cn, cxm, ctx, sxt, fep	R	S	R	R	S	R	S	R	S	S	S	S	S	R	R	S	S	S	S	S	S			
SD 6/2 –100a	E. coli	sw_16	CTX–M1	am, cn, cxm, ctx, fep, tet, na	R	S	R	R	S	R	S	S	S	S	S	S	S	S	R	S	S	S	R	R	S			
SD 6/2 –100d	E. coli	sw_17	CTX–M1	am, cn, cxm, ctx, fep, tet, na	R	S	R	R	S	R	S	S	S	S	S	S	S	R	R	S	S	S	R	R	S			
SD 6/4 –100a	E. coli	sw_18	CTX–M14	am, cn, cxm, ctx, tet, na	R	S	R	R	S	R	S	S	S	S	S	S	S	S	S	S	S	S	R	R	S			
SD 6/4 –100c	E. coli	sw_19	CTX–M14	am, cn, cxm, ctx, fep, tet, na	R	S	R	R	S	R	S	S	S	S	S	S	S	R	R	S	S	S	R	R	S			
SD 10/1–100b	E. coli	sw_20	CTX–M1	am, cn, cxm, ctx, sxt, tet	R	S	R	R	S	R	S	R	S	S	S	S	S	R	R	S	S	S	R	S	S			
SD 10/4–100a	E. coli	sw_21	CTX–M1	am, cn, cxm, ctx, tet	R	S	R	R	S	R	S	S	S	S	S	S	S	S	R	S	S	S	R	S	S			
SD 10/5–100a	E. coli	sw_22	CTX–M1	am, cn, cxm, ctx, gm, sxt, tet, c	R	S	R	R	S	R	R	R	S	S	S	S	S	S	R	S	S	S	R	S	R			
SD 11/4–100a	E. coli	sw_23	CTX–M1	am, cn, cxm, ctx, fep, tet, na	R	S	R	R	S	R	S	S	S	S	S	S	S	R	R	S	S	S	R	R	S			
SD 11/5–100a	E. coli	sw_24	CTX–M1	am, cn, cxm, ctx, fep, tet, na	R	S	R	R	S	R	S	S	S	S	S	S	S	R	R	S	S	S	R	R	S			
SD 15/1–100b	E. coli	sw_25	CTX–M1	am, cn, cxm, ctx, tet	R	S	R	R	S	R	S	S	S	S	S	S	S	R	R	S	S	S	R	S	S			
SD 15/2–100a	E. coli	sw_26	CTX–M1	am, cn, cxm, ctx, fep,tet	R	S	R	R	S	R	S	S	S	S	S	S	S	S	R	S	S	S	R	S	S			
SD 15/3–100a	E. coli	sw_27	CTX–M1	am, cn, cxm, ctx, tet, na	R	S	R	R	S	R	S	S	S	S	S	S	S	S	R	S	S	S	R	R	S			
SD 15/5–100a	E. coli	sw_28	CTX–M1	am, cn, cxm, ctx, fep, tet	R	S	R	R	S	R	S	S	S	S	S	S	S	S	R	S	S	S	R	S	S			
SD 15/6–100a	E. coli	sw_29	CTX–M1	am, cn, cxm, ctx, tet	R	S	R	R	S	R	S	S	S	S	S	S	S	S	R	S	S	S	R	S	S			
SD 15/10–100a	E. coli	sw_30	CTX–M1	am, cn, cxm, ctx, fep	R	S	R	R	S	R	S	S	S	S	S	S	S	R	R	S	S	S	S	S	S			
HD 1/1 100a Th	E. coli	bs_31	SHV–12	am, cxm, ctx, sxt, caz, tet, na, c	R	S	S	R	S	R	S	R	S	S	S	S	S	R	S	S	S	S	R	R	R			
HD 1/1 100b Th	E. coli	bs_32	SHV–12	am, cxm, ctx, sxt, caz, tet, na, c	R	S	S	R	S	R	S	R	S	S	S	S	S	R	S	S	S	S	R	R	R			
HD 1/1 100c Th	E. coli	bs_33	SHV–12	am, cxm, ctx, sxt, caz, tet, na, c	R	S	S	R	S	R	S	R	S	S	S	S	S	R	S	S	S	S	R	R	R			
HD 1/2 100a Th	E. coli	bs_34	CTX–M1	am, cn, cxm, ctx, caz, tet, na, c	R	S	R	R	S	R	S	S	S	S	S	S	S	R	S	S	S	S	R	R	R			
HD 1/2 100b Th	E. coli	bs_35	SHV–12	am, cn, cxm, ctx, caz, tet, na, c	R	S	R	R	S	R	S	S	S	S	S	S	S	R	S	S	S	S	R	R	R			
HD 1/2 100c Th	E. coli	bs_36	CTX–M1	am, cn, cxm, ctx, caz, tet, na, c	R	S	R	R	S	R	S	R	S	R	S	S	S	R	S	S	S	S	R	R	R			
HD 1/2 100d Th	E. coli	bs_37	SHV–12	am, ctx, caz, tet, na, c	R	S	S	S	S	R	S	S	S	R	S	S	S	R	S	S	S	S	R	R	R			
HD 2/9–0a	E. coli	bs_38	SHV–12	am, cxm, ctx, caz, tet, na, c	R	S	S	R	S	R	S	S	S	S	S	S	S	R	S	S	S	S	R	R	R			
HD 3/10–0c	E. coli	bs_39	SHV–12	am, sxt, caz, tet, na	R	S	S	S	S	R	S	R	S	S	S	S	S	R	S	S	S	S	R	R	R			
HD 3/2 100a	E. coli	bs_40	SHV–12	am, sxt, caz, tet, na, c	R	S	S	S	S	R	S	R	S	R	S	S	S	R	S	S	S	S	R	R	R			
HD 3/3–100a	E. coli	bs_41	SHV–12	am, sxt, caz, tet, na, c	R	S	S	S	S	R	S	R	S	S	S	S	S	R	S	S	S	S	R	R	R			
HD 3/4–0a	E. coli	bs_42	SHV–12	am, sxt, caz, tet, na, c	R	S	S	S	S	R	S	R	S	S	S	S	S	R	S	S	S	S	R	R	R			
HD 3/5–0a	E. coli	bs_43	SHV–12	am, sxt, caz, tet, na, c	R	S	S	S	S	R	S	R	S	S	S	S	S	R	S	S	S	S	R	R	R			
HD 8/2–100a	E. coli	bs_44	SHV–2	am	R	S	S	S	S	R	S	S	S	S	S	S	S	S	S	S	S	S	S	S	S			
HD 9/2–0b	E. coli	bs_45	SHV–12	am, caz, tet	R	S	S	S	S	R	S	S	S	S	S	S	S	R	S	S	S	S	R	S	R			
HD 9/2–100b	E. coli	bs_46	SHV–12	am, sxt, caz, tet, na	R	S	S	S	S	R	S	R	S	S	S	S	S	R	S	S	S	S	R	R	R			
HD 6/1–1a	E. faecium	bs_47	VanA	am, va	R							S					S									R	S	R
HD 5/3–2a	E. faecium	bs_48	VanA	am, va	R							S					S									R	S	R

[a] SD x/y…intestine of swine, herd number/isolate number, HD x/y…intestine of broiler, herd number/isolate number

[b] sw…intestine sample taken from swine, bs.... intestine sample taken from broiler

[c] am, ampicillin; amc, amoxicillin/clavulanic acid; tzp, piperacillin/tazobactam; cn, cephalexin; cxm, cefuroxime; fox, cefoxitin; ctx, cefotaxime; caz, ceftazidime; fep, cefepime; cip, ciprofloxacin; mxf, moxifloxacin; gm, gentamicin; an, amikacin; sxt, trimethoprim/sulfamethoxazole; cl, clindamycin; tgc, tigecycline; ipm, imipenem; mem, meropenem; te, tetracycline; na, nalidixic acid; c, chloramphenicol; va, vancomycin; lzd, linezolid; tec, teicoplanin.
